# *In Vivo* Time-Related Evaluation of a Therapeutic Neutralization Monoclonal Antibody against Lethal Enterovirus 71 Infection in a Mouse Model

**DOI:** 10.1371/journal.pone.0109391

**Published:** 2014-10-03

**Authors:** Zhiqun Li, Longfa Xu, Delei He, Lisheng Yang, Che Liu, Yixin Chen, James Wai Kuo Shih, Jun Zhang, Qinjian Zhao, Tong Cheng, Ningshao Xia

**Affiliations:** 1 State Key Laboratory of Molecular Vaccinology and Molecular Diagnostics, School of Life Sciences, Xiamen University, Xiamen Fujian, PR China; 2 National Institute of Diagnostics and Vaccine Development in infectious diseases, School of Public Health, Xiamen University, Xiamen Fujian, PR China; University of Georgia, United States of America

## Abstract

Enterovirus 71 (EV71) is a neurotropic virus capable of inducing severe neurological symptoms and death. No direct targeting antivirals are useful in the treatment of severe EV71 infection. Because of low toxicity and good specificity, monoclonal antibodies (MAb) are a potential candidate for the treatment of viral infections. Therefore, we developed an EV71-specific conformational MAb with high *in vitro* cross-neutralization activity to heterologous EV71 subgenotypes. The *in vivo* treatment experiment at different days post-infection indicated that a single treatment of MAb CT11F9 within day 3 post-infection fully protected mice from morbidity and mortality (0% PBS vs. 100% at 10 µg/g per body weight ***P<0.0001). Immunohistochemical and histological analysis confirmed that CT11F9 significantly prohibited EV71 VP1 expression in various tissues and prevented EV71-induced myonecrosis. Moreover, thrice-treatment at day 4, 5, 6 post-infection was associated with an increased survival rate (18.2% single vs. 50% thrice at 20 µg/g per body weight), and the mice recovered from limb paralysis. Competitive ELISA also confirmed that CT11F9-recognized epitopes were immunodominant in humans. In conclusion, MAb CT11F9 is an ideal candidate to be humanized and used in severe EV71 infection.

## Introduction

Enterovirus 71 (EV71), belonging to the genus Enterovirus of the family Piconaviridae, is one of the major causative pathogens of hand, foot and mouth disease (HFMD). Large outbreaks associated with EV71 have been reported worldwide, especially in Asian-Pacific countries, since 1997 [1]–[4]. Although most EV71 infections are self-limited and only cause mild symptoms, severe complications such as acute flaccid paralysis, encephalitis, pulmonary edema and death have been described [5]. EV71 has been regarded as the most important neurotropic enterovirus since the eradication of the poliovirus.

More than 25 MAbs have been approved for clinical use for diseases such as cancer, autoimmunity and inflammation, as well as infectious disease [6]. It is possible that MAb may be an ideal therapy option for EV71 infection. Research has confirmed that a thrice-daily injection of 5.0 mg/mouse of the neutralization polyclonal antibody at 4 h and 1 and 2 days post-infection can fully protect mice from EV71-induced death [7]. Neutralization monoclonal antidody (nMAb) also had treatment effect: It has been shown that an EV71 MAb belonging to isotype IgM can provide 100% *in vivo* protection when it was administered at 10 µg/g of body weigh to 2-week-old AG129 mice, which lack type I and II interferon receptors, one day prior to lethal EV71 challenge [8]. Single-treatment with an EV71-VP1 epitope-targeted nMAb (10 µg/g of body weight) at 1 day post-infection was also effective in preventing EV71-induced morbidity and mortality [9]. In our recent research, we have successfully protected mice from lethal EV71 challenge with an EV71-VP2 targeted nMAb at 1 day post-infection [10]. Although these data provide support for the effectiveness of neutralization antibody in the treatment of EV71 infection at early times of infection, there is a period of time in the host from the time of viral infection to the appearance of symptoms, which is a few days in a mouse model [11]. As reported, effective first-treatment time was limited within 24 h post-infection, and less is known about the comparative effect of different first-treatment times ranging from infection to death. In addition, death after onset of EV71-induced illness that rapidly progressed to severe cardiopulmonary failure has been observed [1], [2], [12], which suggests that it becomes much harder to treat the infection the closer the first-treatment time was to the onset of illness. Therefore, identifying the suitable time to begin antibody therapy and whether antibody therapy is effective in mice with mild or severe complications will provide important information as to the treatment potential of MAb to EV71 infection and will provide guidance for clinical therapeutic usage in EV71 infection.

In a previous study, we characterized a conformational MAb CT11F9 with *in vivo* neutralization activity [13]. In this study, we further characterize the antibody's *in vivo* neutralization ability and competitive ability to human serum. The antibody's treatment effect under different treatment times and treatment frequencies after EV71 infection was tested, which highlighted the treatment potential of the nMAb in severe EV71 infection.

## Materials and Methods

### Ethics statement

All animal experiments were carried out in accordance with the guidelines of the Xiamen University Institutional Committee for the Care and Use of Laboratory Animals and were approved by the Xiamen University Laboratory Animal Management Ethics Committee. Written informed consent was obtained from the donor for use of the serum sample. Independent Ethics Committee approval was obtained from the Ethics Committee of the National Institute of Diagnostics and Vaccine Development in infectious diseases.

### Cells and viruses

Human muscular rhabdomyosarcoma (RD) cells were obtained from the American Type Culture Collection (ATCC) and cultivated in Minimal Essential Medium (MEM, GIBCO) supplemented with 10% FBS (GIBCO) plus L-glutamine, penicillin, and streptomycin. Eight EV71 clinical isolates were used (Table 1). Five Taiwan isolates and one prototype strain, BrCr/USA/1970, were sourced from the National Taiwan University; one genotype B3 strain, SK-EV006/Malaysia/1997, was sourced from the Tokyo Metropolitan Institute for Neuroscience of Japan; and the EV71/Jiangsu/2008 was isolated in Jiangsu Province. A mouse-adapted virus named pSVA-MP4 was generated by four passages in newborn mice using SK-EV006/Malaysia/1997. The EV71 virus was loaded onto a 15–50% continuous sucrose gradient, resulting in fractions with densities at 20–40% after 3 h of ultracentrifugation (32,000×g, SW41Ti rotor, Beckman). The fractions were collected, pelleted (100,000×g for 2 h) and resuspended in PBS. The protein content of the purified virus was measured by the BCA protein assay (Bio-Rad), and the samples were then stored in a −80°C freezer.

**Table 1:** Representative strains of EV71 subgenotypes.

GenBank No.	Strain name	Abbreviation	Genotype
U22521	BrCr/USA/1970^a^	BrCr	EV71 A
AB469182	SK-EV006/Malaysia/1997	EV006	EV71 B3
JF420549	02203/Taiwan/2002	02203	EV71 B4
JF420553	03315/Taiwan/2008	03315	EV71 B5
JF420552	03149/Taiwan/2008	03149	EV71 C2
FJ600325	EV71/Jiangsu/2008	Jiangsu	EV71 C4
JF420554	02969/Taiwan/2008	02969	EV71 C5
JF420555	00190/Taiwan/2008	00190	CA16^b^

a Isolation place and isolation year are shown in the name of each virus.

b CA16, coxsackie virus A 16.

### Indirect immunofluorescence assay

RD cells were seeded overnight onto 24-well plates and then infected with EV71 Jiangsu. After incubating at 37°C for 12 h, the cells were fixed with 4% paraformaldehyde/PBS for 30 min followed by permeabilizing with 0.1% Triton/PBS for 10 min. The cells were blocked with 4% FBS/PBS for 2 h at 37°C, incubated with MAb CT11F9 solution for 1 h at 37°C, and then incubated with FITC-coupled secondary antibodies for 30 min at 37°C, followed by incubating with DAPI for 5 min at room temperature. The cells were washed in PBS three times every 5 min. The results were observed using a Laser Scanning Confocal Microscope (MRC-1024, Biorad, Hercules, CA) with ZEN software.

### Competitive ELISA

Human serum was collected from HFMD patients between March and September 2008 during an outbreak of HFMD (Table 2). The clinical human serum samples used for this study were well characterized for the viral RNA and EV71 specific neutralization antibody titers in our previous work [14], [15]. The 96-well plates were coated at 4°C overnight with EV71 Jiangsu in 50 mM carbonate buffer (pH 9.6). After washing with PBST (0.05% Tween 20 in PBS), the plates were blocked with 1% bovine serum albumin (BSA) in PBS at 37°C for 2 h. MAb CT11F9 (10 µg per well) or an well charactered anti-CB3 MAb A13H11 [16] (10 µg per well) was added and the plates were incubated for 30 min at 37°C. Human serum was added to the plates (1∶50 dilution) and incubated at 37°C for 30 min. Mouse anti-human antibody labeled with horseradish peroxidase was added to the plates at a 1∶200 dilution in PBS with 1% BSA, and incubated for 30 min at 37°C. The wells were washed 5 times with PBST between each step. Visualization was achieved by incubation with o-phenyl-diamine-2HCl for 15 min, and the reaction was stopped by adding 50 µl of 2 M H_2_SO_4_. The OD value (A_450/620_) was converted to percentage inhibition (PI) using the formula: PI (%) = 100−[(OD_sample_/OD_control_)×100]. The OD_control_ represents the well containing human serum alone.

**Table 2:** Information from the human serum samples collected from HFMD patients.

	PCR Assay		Neutralization Titers	
Sera No.	CA16	EV71	CA16	EV71
1	−	+	0	1024
2	−	+	4096	1024
3	−	+	0	256
4	−	+	0	512
5	−	+	32	256
6	−	+	0	2048
7	−	+	0	2048
8	−	+	0	2048

### *In vitro* neutralization assay

RD cell monolayers were prepared by seeding at 1,000 cells per well into 96-well plates. Two-fold serial dilutions of MAb CT11F9 (1 mg/ml) were prepared in MEM. Each sample was challenged with 100 TCID_50_ EV71 strains shown in Table 1. After incubation at 37°C, 5% CO_2_, for 1 h, the serially diluted samples were incubated with RD cells prepared in 96-well plates and incubated at 37°C for 7 days. The neutralization titers were read as the highest dilution in over 50% CPE. Each assay was processed independently three times.

### *In vivo* treatment of mice

Inbred pregnant BALB/c mice were obtained from the Slac Laboratory Animal Co., Ltd., Shanghai, China. The newborn mice were challenged intraperitoneally (i.p.) with 50 µl of EV71 (10^7^ TCID_50_ per mouse), followed by i.p. inoculated with 50 µl of MAb CT11F9. For the single treatment, CT11F9 (10 µg/g per body weight) was given at day 1, 2, 3, 4, or 5 post-infection. For the thrice-treatment, CT11F9 (20 µg/g per body weight per day) was given at day 4, 5, 6 or 5, 6, 7 post-infection. The mice in the control groups were treated with 50 µl of PBS. Every group (n = 10–11) contained two independent experiments. The mice were monitored daily for body weight, clinical illness and death until day 20 post-infection. The grade of clinical disease was scored as follows: 0, healthy; 1, lethargy and inactivity; 2, wasting; 3, limb weakness; 4, hind limb paralysis; and 5, moribund and death.

### Histology and immunohistochemistry analysis

Another two groups of newborn mice (6 newborn mice per group) were challenged i.p. with 50 µl of EV71 (10^7^ TCID_50_ per mouse). At day 3 post-infection, one group was given 50 µl of CT11F9 (10 µg/g per body weight) per mouse, the other group was given 50 µl of PBS per mouse. Eight days after infection, the mice were euthanized using carbon dioxide and subjected to histopathological and immunohistochemical (IHC) examination. Brain, spinal cord, heart, liver, lung, intestines and limb muscles were separately harvested, and then fixed by immersion in 4% formalin/PBS for 72 h at room temperature. The fixed tissues were bisected, embedded in paraffin and sectioned (4 mm). For histopathological analysis, tissue sections were stained with hematoxylin and eosin (HE). IHC examinations were performed using an UltrasensitiveTM S-P kit (Fuzhou Maixin Biotechnology Development Co., Ltd., Fuzhou, China) and DAB Detection Kit (Streptavidin-Biotin; Fuzhou Maixin Biotechnology Development Co., Ltd., Fuzhou, China) according to the manufacturer's recommendations. The primary antibody, I2D7, was a mouse anti-EV71 VP1 MAb (1 mg/ml, 1∶1,000 dilution).

### Statistical analysis

The survival curves were evaluated by the Mantel–Cox Log-rank test. The inhibition rate of the competitive ELISA was evaluated by the paired t-test. The health scores were shown as means. The competitive ELISA were shown as means +/−standard deviations (SD). A P value of <0.05 was considered as statistical significance.

## Results

### *In vitro* cross-neutralizing ability of monoclonal antibody CT11F9

**Figure 1:**
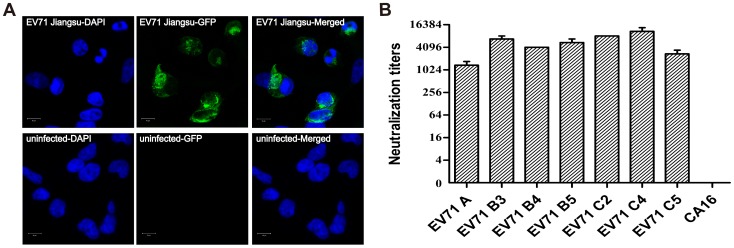
Analysis of CT11F9 by immunofluorescence and an *in vitro* neutralization test. (**A**) Immunofluorescence images of CT11F9 against EV71 Jiangsu-infected RD cells or uninfected RD cells are shown as DAPI, GFP and a merged image of DAPI and GFP, respectively. (**B**) Neutralization titers of MAb CT11F9 against seven EV71 subgenotypes and CA16.

In a recent study, we identified a series of nMAbs that could not react with whole purified EV71 Jiangsu or VP1, VP2 and VP3 proteins of EV71 by western blotting, which indicated that they recognized conformational epitopes of EV71 [13]. Conformational MAb CT11F9, which had the highest *in vitro* neutralization titer, was selected to be further characterized. The laser-confocal results showed that CT11F9 specifically bound parental EV71 Jiangsu, mainly in the cytoplasm of RD cells (Fig. 1A). Detailed *in vitro* cross-neutralizing titers against seven EV71 strains covering the A, B3, B4, B5, C2, C4 and C5 subgenotypes showed that neutralization titers of more than 1∶1028 were reached (Fig. 1B). According to these results, we felt that CT11F9 was a good candidate for testing in animals.

### *In vivo* single-treatment of CT11F9 in a time-related manner

**Figure 2:**
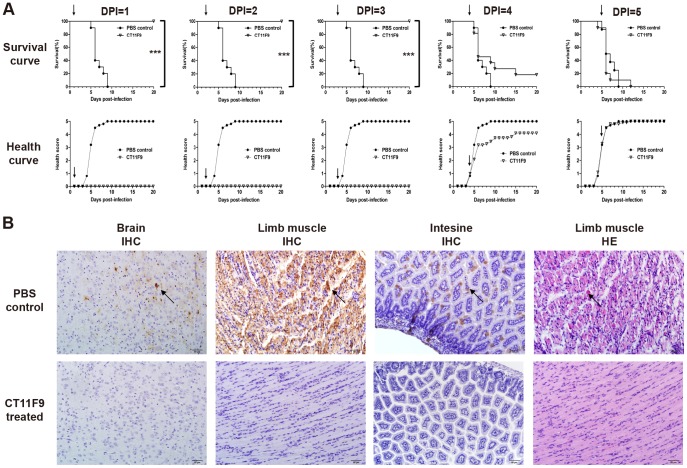
*In vivo* single-treatment of CT11F9 at different times after infection with EV71. (**A**) Kaplan-Meier survival curve and health scores of newborn BALB/c mice i.p. challenged with 10^7^ TCID_50_ pSVA-MP4 and then treated with MAb CT11F9 at different days post-infection (DPI). Deaths were scored as 5 until the end of the experiment; the treatment time is indicated by the black arrow. (**B**) Brain, limb muscle and intestine were analyzed by IHC; limb muscle was also analyzed by HE staining. Positive reactions are indicated by the black arrow. Representative images are shown at a magnification of 200X. The asterisks indicates significant differences of ***P<0.0001.

To identify the influence of different treatment time on the morbidity and mortality of mice, groups of newborn BALB/c mice were infected i.p. with 10^7^ T CID_50_ of pSVA-MP4, followed by injection i.p. with CT11F9 at day 1, 2, 3, 4 or 5 post-infection (10 µg/g of body weight, single-treatment). The control mice that received PBS developed illness as early as day 4 post-infection and progressed to limb paralysis from day 5 post-infection. The control mice died within day 9 post-infection, while all the mice that received CT11F9 by day 3 post-infection were healthy throughout the experiment. However, a single treatment at day 4 or 5 post-infection did not show a significant treatment effect (Fig. 2A).

To confirm the treatment effect of CT11F9 at day 3 post-infection, HE staining and IHC examinations were undertaken. The IHC results revealed the EV71-VP1 antigen in the medulla oblongata of the brain, and in limb muscles and intestines of PBS-treated mice, in contrast to CT11F9-treated mice (Fig. 2B). Severe necrotizing myositis was also observed in the limb muscles of PBS-treated mice compared to the normal morphology of CT11F9-treated mice by HE staining (Fig. 2B). These results show that a single treatment of MAb CT11F9 was able to fully protect mice from EV71-induced morbidity and mortality within day 3 post-infection, which was very close to the time when the mice began to present visible symptoms.

### *In vivo* thrice-treatment of CT11F9

**Figure 3:**
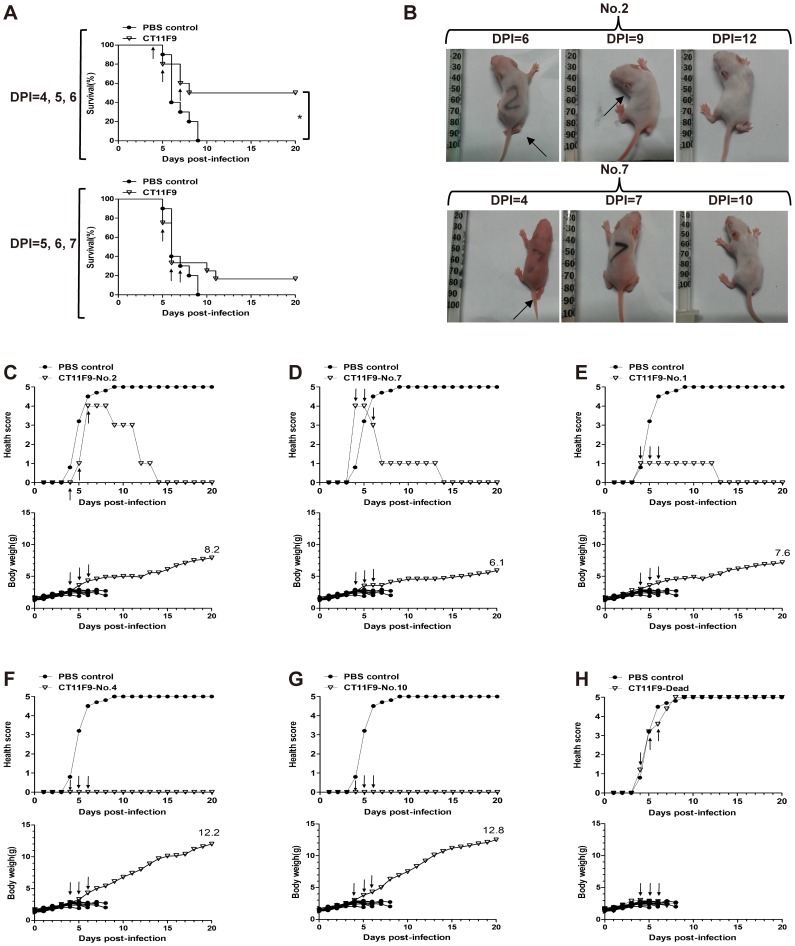
*In vivo* thrice-treatment with CT11F9. (**A**) Kaplan-Meier survival curve of newborn BALB/c mice (n = 10) i.p. challenged with 10^7^ TCID_50_ pSVA-MP4 and treated with MAb CT11F9 at days 4, 5, 6 or 5, 6, 7 post-infection. (**B**) Photographs of two mice recovered from severe limb paralysis from the day 4, 5, 6-treated group were taken on day 6, 9, 12 (No. 2) or 4, 7, 10 (No. 7) post-infection. The black arrow indicates limb paralysis (No. 2, DPI = 9; No. 7, DPI = 4) or curved body shape (No. 2, DPI = 9). (**C-H**) Health scores and body weight of mice from the day 4, 5, 6-treated group. In this group, (**C-E**) three mice recovered from illness, (**F,G**) two mice showed no symptoms, (**H**) the remaining five mice died. Deaths were scored as 5 until the end of the experiment and the body weight of each mouse is shown. The treatment time is indicated by the black arrow. The asterisks indicate significant differences of *P<0.05.

To achieve a significant treatment effect in morbid mice, an increase in treatment dose and frequency might be needed. For this reason, a continuous thrice-daily treatment on day 4, 5, 6 or day 5, 6, 7 post-infection (20 µg/g of body weight per day) was performed. Treatment begun at day 5 post-infection did not improve the survival rate, while treatment begun at day 4 post-infection increased the survival rate to 50% (Fig. 3A). Among the five surviving mice, two mice were healthy throughout the experiment (Fig. 3F and G). Three mice showed symptoms: two mice gradually recovered from limb paralysis, limb weakness or a curved body shape, lethargy and inactivity to normal status starting from day 4 or day 6 post-infection (Fig. 3B, C and D); the other mouse only showed lethargy and inactivity (Fig. 3E). These three mice had significantly lower body weights compared to the normal surviving mice in the same group (Fig. 3C, D, E, F and G). The remaining five mice died, similarly to the control group (Fig. 3H). Our results suggested that even when visible severe symptoms appeared, MAb CT11F9 had the ability to cure the illness, although some sequelae might occur.

### Competitive ELISA of CT11F9 against human sera

**Figure 4:**
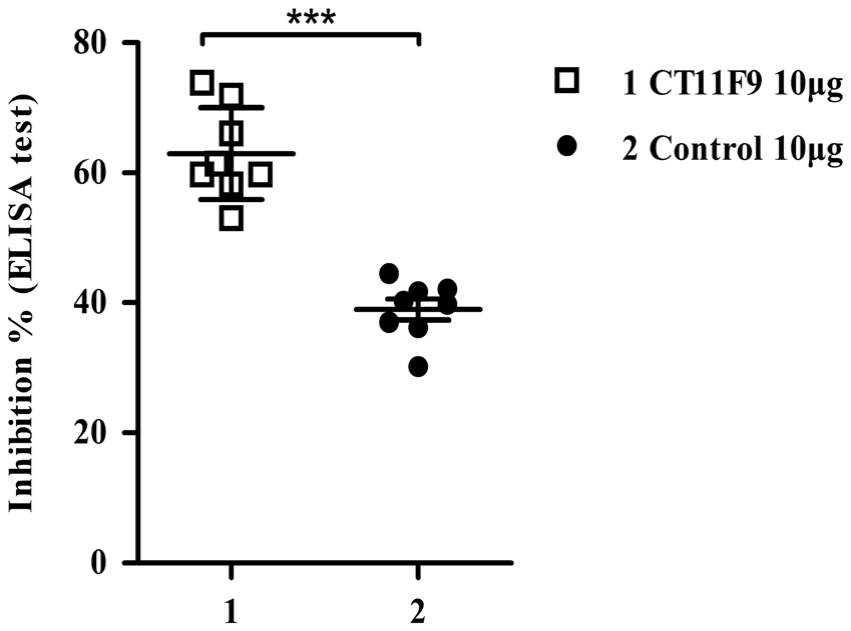
Competitive ELISA of CT11F9 against human serum. The MAb CT11F9 (10 µg/g) competed with eight human serum samples for specifically binding to EV71 Jiangsu coated on the well. The irrelevant MAb control was a CB3-VP1 targeted MAb. The MAb CT11F9 blocked 63% of EV71-infected human sera from binding EV71 Jiangsu. The asterisks indicates significant differences of ***P<0.0005.

Protection in mice may not reflect a protective effect in humans. To further explore the potential of MAb CT11F9 in clinical therapy for human infection of EV71, eight human serum samples from EV71 infected patients confirmed by RT-PCR and neutralization assays were used to investigate the abundance of CT11F9-recognized epitopes to human serum by competitive ELISA. By calculating the mean PI values, the MAb CT11F9 have been shown to block 63% of EV71-infected human sera from binding EV71 Jiangsu, while the control group using an irrelevant anti-CB3 MAb showed 39% inhibition (Fig. 4). The significant difference of PI values (***P<0.0005) between CT11F9 and control indicated that CT11F9 recognized immunodominant epitopes in humans and is of potential in clinical therapy for EV71 infection.

## Discussion

EV71-induced severe complications and death make it urgent to develop effect antiviral drugs to prevent EV71-induced morbidity and mortality. Although increasing numbers of anti-EV71 drugs have been reported, including small molecules and non-conventional nucleic-acid-based strategies [17], additional rigorous animal experiments or clinical tests are needed to determine their safety.

Neutralization antibodies play an important defensive role in viral infection. Although *in vivo* protection is complex because it involves the interaction of antibodies with cells and molecules of the innate immune system, most neutralization antibodies identified under defined experimental conditions also protect animals [18]. For example, passive transfer of specific nMAbs reduces virus-induced severity of Venezuelan equine encephalitis virus [19], West Nile virus (WNA) [20] and influenza virus [21], [22]. In this study, we attempted to evaluate the potential of a neutralization antibody to treat EV71 infection and to identify the suitable time that antibody therapy may begin using a time-related treatment protocol in a mouse model, with different treatment times between the EV71 challenge to EV71-induced severe illness, which we thought was an important index for therapeutic antibodies. An optimal single-treatment time within day 3 post-infection was identified, after which the mice began to show visible illness. This was evidenced by the IHC and HE results from the brain, limb muscle and intestine in antibody-treated mice because EV71 may spread into the CNS through neural pathways to induce severe CNS complications [23], [24]. These data supported the observation that injection of CT11F9 within day 3 post-infection effectively neutralized the virus *in vivo* and prevented the spread of EV71 into muscle tissues and CNS. Because antibodies cannot enter the cell cytoplasm, once the virus widely infects the cells of the CNS and limb muscle to cause tissue damage, most antibodies will not be able to clear the virus and reverse these symptoms. This may explain why single-treatment with CT11F9 failed to offer a noticeable treatment effect at day 4 or 5 post-infection when the challenged mice began to have slight complications or even paralysis. However, a thrice-treatment protocol with CT11F9 (20 µg/g per body weight) from day 4 post-infection successfully achieved a 50% survival rate, and the mice recovered from inactivity or even paralysis. These results indicate that optimal EV71 antibody therapy should be started before visible illness is observed and that a continuous treatment with a higher dose is needed once illness has occurred.

The lack of a suitable EV71 animal model has limited the understanding of EV71 pathogenesis and the development of EV71 therapeutic MAbs. Considering the aspects of ethics, economy and infectivity, a mouse model is more suitable for EV71 research and can be useful for seeking potential therapeutic MAbs although we have no ideal about the similarity of the EV71 progression between mouse and human. The *in vivo* result have showed that the MAb CT11F9 was of good treatment effect in mouse model. Nonetheless, it is important and necessary to humanized the MAb CT11F9 and evaluate it's treatment effect in a more suitable animal model when it becomes possible.

Anti-inflammatory MAbs is willing to be a good therapeutic choise for EV71 infection. Besides the virus-induced pathogenicity, it has been proposed that the induction of proinflammatory cytokines is responsible for the pathogenicity of EV71 [25]–[27]. Animal experiments have also confirmed that the injection of anti-IL-6 antibodies increases the survival rate of EV71-infected mice [28]. Considering of this, EV71 therapy is not only clearance of the virus but also restoration of the body immune reaction. Thus, combinations of anti-virus nMAbs with anti-inflammatory MAbs may be a good choice to be tested for further animal treatment experiments.

In conclusion, based on the *in vitro* cross-neutralization ability against different EV71 subgenotypes and the *in vivo* treatment effect in morbid mice, an EV71-specific nMAb CT11F9 that recognized immunodominant epitopes in humans is a promising candidate to be humanized and used in clinical therapy for EV71 infection.
